# Severe Transfusion-Dependent Infantile Hemolytic Anemia Associated With a Novel Homozygous PKLR Variant of Uncertain Significance (c.1376C>G, p.Ala459Gly) and Coexisting G6PD Deficiency: A Case Report

**DOI:** 10.7759/cureus.113577

**Published:** 2026-07-29

**Authors:** Sara S Hassanien, Badriah G Alasmari, Ehab Hanafy, Mohamed Mansour, Ahlam Babiker

**Affiliations:** 1 Department of Pediatric Hemato-Oncology, Armed Forces Hospital Southern Region, Khamis Mushait, SAU; 2 Department of Pediatric Critical Care Medicine, Armed Forces Hospital Southern Region, Khamis Mushait, SAU

**Keywords:** chronic hemolytic anemia, g6pd deficiency, iron overload, novel mutation, pyruvate kinase deficiency

## Abstract

Pyruvate kinase deficiency (PKD) results from pathogenic variants in the PKLR gene and demonstrates marked clinical heterogeneity ranging from compensated hemolysis to severe transfusion-dependent anemia; however, novel variants continue to expand the genotypic spectrum of the disease.

We report a case of a 10-month-old girl with transfusion-dependent chronic hemolytic anemia found to harbor a novel homozygous PKLR missense variant c.1376C>G (p.Ala459Gly). This variant has not previously been reported in the literature and was classified as a variant of uncertain significance (VUS) according to the American College of Medical Genetics and Genomics/Association for Molecular Pathology (ACMG/AMP) criteria.

This report expands the mutational spectrum of PKD and highlights the importance of molecular testing in infants with unexplained chronic hemolytic anemia, especially in populations with high consanguinity rates. Although functional validation of the identified PKLR variant was not available, the patient's clinical and hematologic phenotype was highly consistent with severe PKD, supporting the variant as a candidate disease-associated alteration.

## Introduction

Pyruvate kinase deficiency (PKD) is a rare autosomal recessive enzymatic disorder and the most common cause of hereditary non-spherocytic hemolytic anemia [1]. It results from mutations in the PKLR gene located on chromosome 1q21, which encodes the red blood cell and liver isoforms of pyruvate kinase, a key regulatory enzyme in glycolysis [1,2]. Pyruvate kinase catalyzes the conversion of phosphoenolpyruvate to pyruvate with the generation of adenosine triphosphate (ATP), a critical energy source for erythrocytes that lack mitochondria [1,3].

The global prevalence of PKD is estimated to be between one in 100,000 and one in 300,000 individuals, although this may be underestimated due to underdiagnosis [2,4]. Clinical manifestations are highly variable, ranging from mild compensated hemolysis to severe neonatal anemia, hydrops fetalis, and transfusion-dependent states [3,5]. Chronic hemolysis may result in multiple complications, including gallstones, splenomegaly, pulmonary hypertension, extramedullary hematopoiesis, and iron overload [4,5].

More than 200 pathogenic variants in PKLR have been identified, with missense mutations representing the majority [2,3]. Common variants include p.Arg510Gln and p.Arg486Trp; however, increasing use of next-generation sequencing has led to identification of numerous rare and novel variants, expanding the genotypic spectrum of the disease [2,6]. In this report, we describe a severe infantile presentation of PKD associated with a novel homozygous PKLR variant c.1376C>G (p.Ala459Gly). At the time of manuscript preparation, the variant was not identified in ClinVar, gnomAD, Leiden Open Variation Database (LOVD), IthaGenes, or the published literature, supporting its rarity and novelty. This case expands the mutational spectrum of PKLR and contributes to the understanding of genotype-phenotype correlations in PKD.

## Case presentation

The patient was a 10-month-old female infant, born to a consanguineous marriage at 37 weeks of gestation via elective cesarean section following an in vitro fertilization (IVF) pregnancy after 10 years of infertility. Her birth weight was 1.6 kg, consistent with intrauterine growth restriction and low birth weight status. Her Apgar scores were 7 and 8 at one and five minutes, respectively.

**Figure 1:**
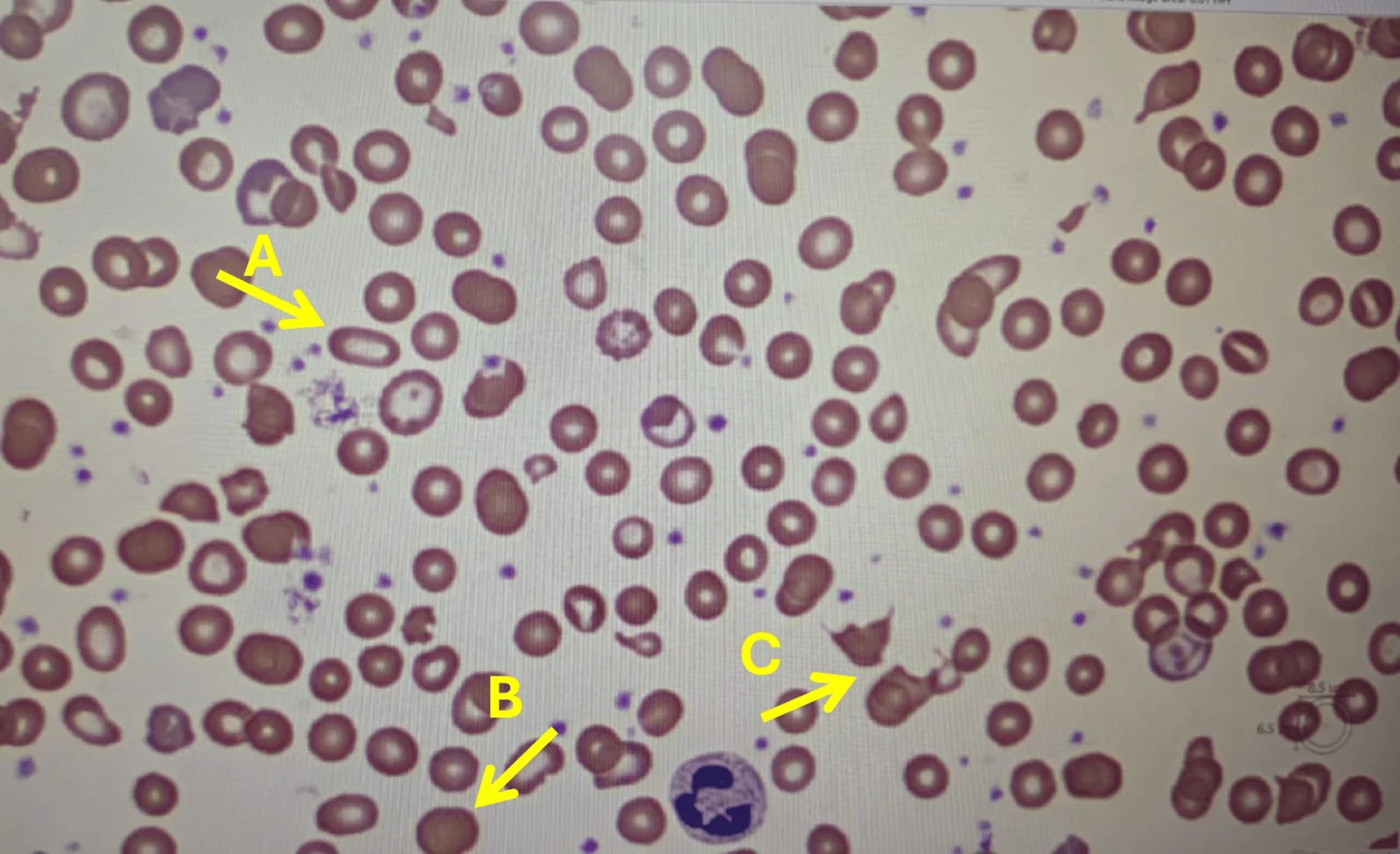
Peripheral blood smear demonstrating anisopoikilocytosis, macrocytosis, and polychromasia. Wright-Giemsa stain, original magnification ×1000 (oil immersion). Scale bar = 10 μm. (A) Anisopoikilocytosis, reflecting variation in red blood cell size and shape associated with chronic hemolysis. (B) Macrocytosis, consistent with an increased proportion of circulating reticulocytes. (C) Prominent polychromasia, indicative of reticulocytosis and compensatory erythropoiesis in response to hemolysis.

Shortly after birth, she developed severe neonatal jaundice and pallor requiring admission to the neonatal intensive care unit. Initial laboratory evaluation demonstrated significant hemolytic anemia with a hemoglobin level of 9.3 g/dL, marked reticulocytosis, macrocytosis, polychromasia, and numerous nucleated red blood cells on peripheral smear (Figure 1).

The direct antiglobulin test was negative, reducing the likelihood of immune-mediated hemolysis and supporting a non-immune hemolytic process. Glucose-6-phosphate dehydrogenase (G6PD) screening revealed a low level of 0.4 U/g Hb (reference range: 4.6-13.5 U/g Hb), which was done after blood transfusion, raising the possibility of coexisting G6PD enzyme deficiency.

Given the rapidly ongoing hemolysis, a single empiric dose of intravenous immunoglobulin in the NICU was administered alongside intensive phototherapy and packed red blood cell transfusion. Cranial and abdominal ultrasonography were unremarkable. TORCH (toxoplasmosis, other infections, rubella, cytomegalovirus, and herpes simplex virus) screening was negative.

She remained hospitalized for 20 days with gradual weight gain from 1.6 kg to 1.8 kg before discharge on folic acid supplementation and hematology follow-up. She was subsequently admitted more than 10 times because of progressive pallor. Her hemoglobin level fluctuated between 6.8 g/dL and 7.5 g/dL (reference range: 10.5-13.5 g/dL for infants), necessitating multiple subsequent blood transfusions (Table 1).

**Table 1:** Chronological summary of the patient's clinical course, laboratory findings, and management. Chronological summary of the patient's clinical course from birth to 10 months of age, including major clinical events, laboratory findings, molecular diagnosis, and therapeutic interventions. Abbreviations: Hb, hemoglobin; G6PD, glucose-6-phosphate dehydrogenase; NICU, neonatal intensive care unit; PRBC, packed red blood cells; PKLR, pyruvate kinase liver/red blood cell gene. * Measured after packed red blood cell transfusion.

Age/Time point	Clinical course and key findings	Management
Birth	Severe neonatal jaundice and hemolytic anemia. Hb = 9.3 g/dL; total bilirubin = 200 μmol/L; reticulocyte count = 36.1%.	NICU admission, intensive phototherapy, first PRBC transfusion.
Day 1	Post-transfusion: Hb = 14.1 g/dL; total bilirubin = 167 μmol/L; G6PD activity = 19.4 U/g Hb*.	Supportive care and clinical monitoring.
Day 3	Recurrent severe anemia (Hb = 7.4 g/dL).	Second PRBC transfusion.
Day 10	Persistent hemolytic anemia despite transfusions. Hb = 6.5 g/dL; G6PD activity = 0.4 U/g Hb.	Third PRBC transfusion and extended diagnostic evaluation.
1-6 months	Chronic transfusion-dependent hemolytic anemia. Pre-transfusion Hb = 6.8-7.5 g/dL.	PRBC transfusions every 3-4 weeks and folic acid supplementation.
7 months	Serum ferritin = 900 ng/mL.	Continued PRBC transfusions.
8 months	Serum ferritin = 974 ng/mL.	Continued PRBC transfusions.
9 months	Serum ferritin = 1,200 ng/mL, consistent with progressive transfusional iron overload.	Continued transfusions with close iron monitoring.
10 months	Whole-exome sequencing identified a homozygous *PKLR* variant (NM_000298.5:c.1376C>G; p.Ala459Gly), classified as a variant of uncertain significance (Class III). Serum ferritin remained >1,200 ng/mL.	Approximately 12 PRBC transfusions were administered; continued hematology follow-up and evaluation for iron chelation therapy.

Her serum ferritin level steadily increased to more than 1200 ng/mL (reference range: 7-140 ng/mL for infants), giving rise to serious concern for secondary iron overload. Cardiac and hepatic T2* MRI were not performed because of the patient's young age and the anticipated requirement for deep sedation. Clinical examination revealed no hepatomegaly, and the spleen was just palpable.

Whole-exome sequencing subsequently identified a homozygous PKLR missense variant, c.1376C>G (p.Ala459Gly), located in exon 10, of uncertain significance according to the American College of Medical Genetics and Genomics/Association for Molecular Pathology (ACMG/AMP) criteria (Table 2) [7].

**Table 2:** Genetic characterization of the identified PKLR variant. Abbreviations: LOVD, Leiden Open Variation Database; ACMG, American College of Medical Genetics and Genomics; AMP, Association for Molecular Pathology; SIFT, Sorting Intolerant From Tolerant.

Parameter	Variant details & specifications	Description/Clinical significance
Gene	PKLR (Pyruvate Kinase L/R)	Encodes the erythrocyte pyruvate kinase enzyme involved in glycolysis.
RefSeq transcript	NM_000298.5	Reference transcript used for clinical annotation.
Exon location	Exon 10	Located within the coding region of PKLR.
Nucleotide alteration	c.1376C>G	Missense nucleotide substitution.
Protein change	p.(Ala459Gly)	Alanine substituted by glycine at codon 459.
Zygosity	Homozygous	Consistent with autosomal recessive inheritance.
Population databases	Absent from gnomAD and the 1000 Genomes Project	Supports rarity of the variant.
Clinical variant databases	Not identified in ClinVar, LOVD, or IthaGenes at the time of manuscript preparation	Supports the novelty of the variant.
In silico predictions	PolyPhen-2: Possibly damaging; SIFT: Deleterious; MutationTaster: Disease causing; Conservation: High	Multiple computational tools predict a deleterious effect on protein function.
Laboratory classification	Variant of uncertain significance (Class III)	Classified according to ACMG/AMP guidelines by the reporting laboratory (Centogene).

As erythrocyte pyruvate kinase enzyme activity testing was unavailable at our institution and recent transfusions would likely have produced unreliable enzyme measurements, diagnosis relied on the compatible clinical phenotype together with molecular findings. The patient continues regular transfusion therapy and folic acid supplementation with close outpatient visits with monitoring of growth, pre-transfusion hemoglobin, and iron status.

## Discussion

**Figure 2:**
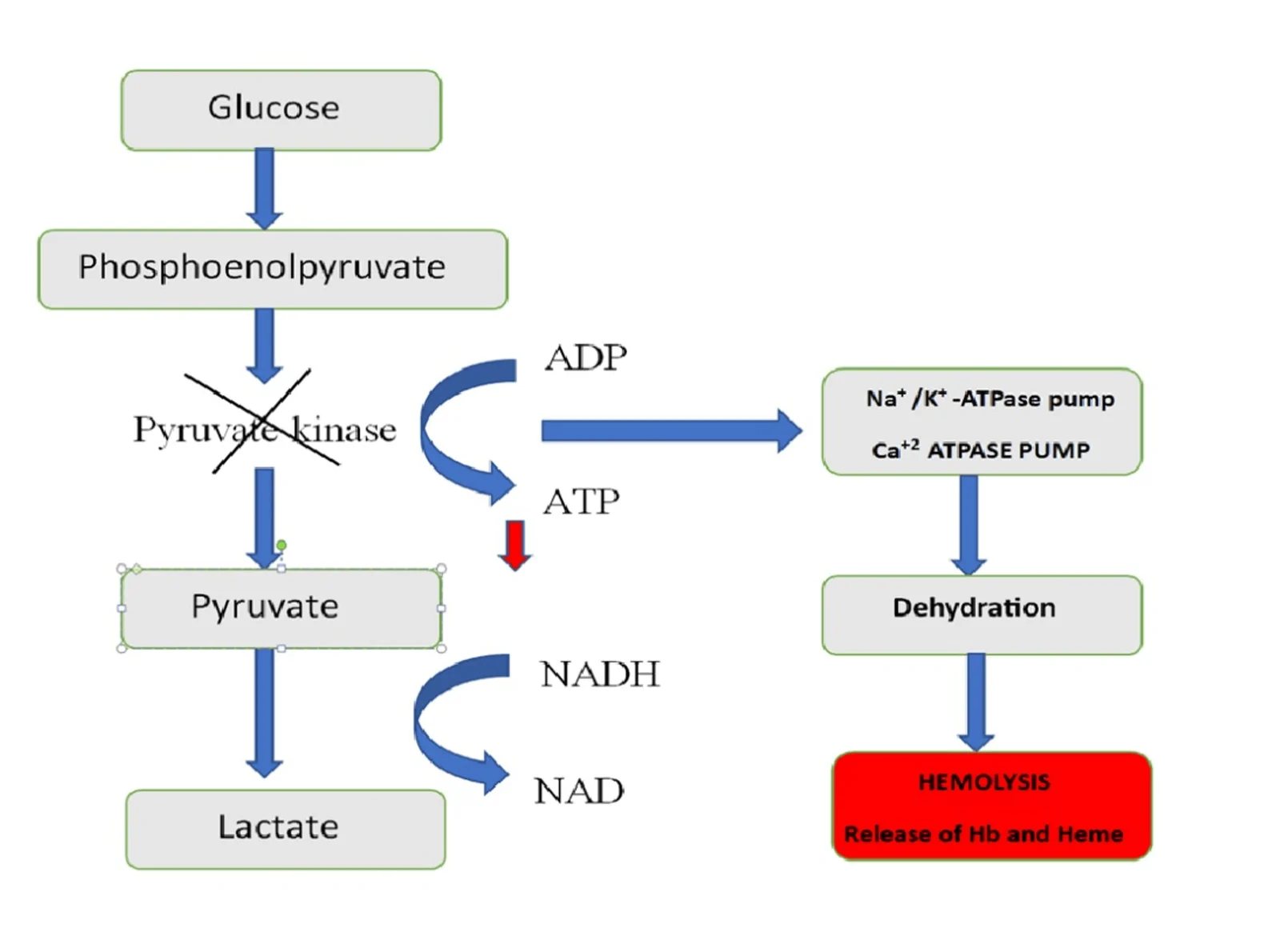
Schematic illustration of the pathophysiology of pyruvate kinase deficiency. Pyruvate kinase catalyzes the conversion of phosphoenolpyruvate to pyruvate, generating ATP during glycolysis. Deficiency of pyruvate kinase reduces ATP production, impairing the activity of ATP-dependent membrane ion pumps, including the Na⁺/K⁺-ATPase and Ca²⁺-ATPase. The resulting loss of membrane ion homeostasis leads to erythrocyte dehydration, decreased red blood cell deformability, and premature destruction of erythrocytes, causing chronic hemolysis. This figure was created by the authors. Abbreviations: ATP, adenosine triphosphate; ADP, adenosine diphosphate; NAD, nicotinamide adenine dinucleotide; NADH, nicotinamide adenine dinucleotide hydrogen; Hb, hemoglobin.

PKD remains a rare but clinically significant cause of hereditary hemolytic anemia, with considerable variability in disease severity depending on the underlying mutation and residual enzyme activity [2,3]. Patients may present with a broad clinical spectrum ranging from mild anemia to severe transfusion-dependent disease, as demonstrated in our case [4]. The pathophysiology of PKD is directly related to impaired ATP production in erythrocytes (Figure 2), leading to membrane instability, cellular dehydration, and premature destruction of red blood cells, primarily in the spleen [1,4]. This energy deficit underlies the chronic hemolysis observed in affected individuals.

Prenatal manifestations of severe PKD include fetal anemia, hydrops fetalis, and prematurity, occasionally necessitating intrauterine transfusion [5]. Although the 2024 international guidelines for the diagnosis and management of PKD do not recognize intrauterine growth restriction (IUGR) as a defining feature of PKD due to insufficient causal evidence [5], its coexistence with early-onset anemia in our patient raises the suspicion of an association. However, in the absence of detailed prenatal assessment, a definitive causal link cannot be established.

Our patient harbored a homozygous PKLR variant, NM_000298.5:c.1376C>G (p.Ala459Gly), which, to the best of our knowledge, had not been previously reported in ClinVar, LOVD, IthaGenes, gnomAD, the 1000 Genomes Project, or the published literature at the time of manuscript preparation. The reporting laboratory classified the variant as a variant of uncertain significance (Class III) according to ACMG/AMP criteria [7]. Nevertheless, the severe clinical phenotype, homozygous inheritance, absence of an alternative molecular diagnosis, and concordant in silico predictions raise the possibility that this variant contributes to disease; however, functional studies and additional unrelated cases are required before pathogenicity can be established. Similar observations have been reported with other novel PKLR variants. Fawaz et al. described a homozygous p.Ala300Pro mutation in an Omani child presenting with severe transfusion-dependent hemolytic anemia [6]. These findings highlight the expanding molecular heterogeneity of PKD and the importance of genetic testing in atypical or severe cases.

An important feature in our patient was the initial biochemical finding of reduced G6PD enzyme activity. Dual erythrocyte enzymopathies can exacerbate hemolysis and contribute to increased disease severity due to heightened oxidative stress [8]. Although G6PD deficiency is an X-linked disorder where female expression can occasionally occur via skewed X-chromosome inactivation or homozygosity [8], whole-exome sequencing in our patient identified no pathogenic G6PD variant. Consequently, PKD accounts for the primary disease course, whereas the reduced G6PD activity (0.4 U/g Hb) represents a secondary biochemical finding.

Consanguinity significantly increases the likelihood of homozygous autosomal recessive disorders, particularly in Middle Eastern populations [9]. This emphasizes the importance of genetic counseling and early diagnostic evaluation in high-risk populations.

Iron overload represents one of the most clinically significant long-term complications of PKD [10]. It may occur even in non-transfused patients due to increased intestinal iron absorption driven by chronic hemolysis and hepcidin suppression [10]. In transfusion-dependent patients, iron accumulation occurs more rapidly, as observed in our patient, necessitating early monitoring and consideration of chelation therapy [4,5,10].

**Figure 3:**
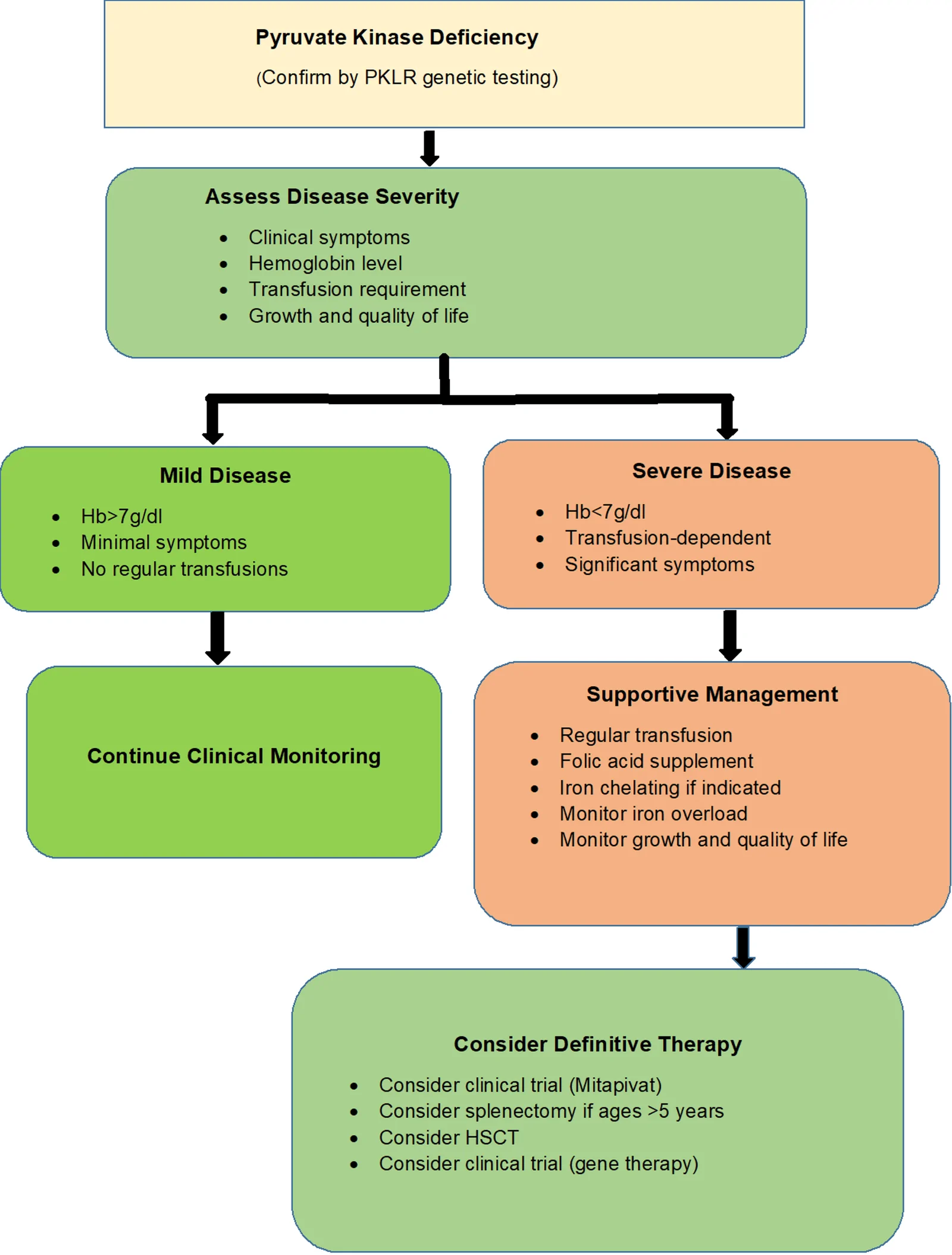
Simplified management algorithm for pyruvate kinase deficiency. Simplified clinical management algorithm for pyruvate kinase (PK) deficiency. Following confirmation of the diagnosis, disease severity is assessed based on clinical symptoms, transfusion requirements, and health-related quality of life. Patients with mild disease are managed with regular clinical monitoring, whereas those with severe disease require supportive care, including red blood cell (RBC) transfusions, folic acid supplementation, iron chelation therapy for iron overload, and consideration of splenectomy in appropriately selected patients. Disease-modifying therapies, including Mitapivat (for adults with suitable PKLR variants) and gene therapy, may be considered in eligible patients. This figure was created by the authors and adapted from Luke et al. [12]. Abbreviations: PK, pyruvate kinase; Hb, hemoglobin; RBC, red blood cell; HSCT, hematopoietic stem cell transplantation.

Historically, management of PKD has been largely supportive and includes red blood cell transfusion, folic acid supplementation, splenectomy in selected cases, and management of iron overload (Figure 3) [4]. Recently, Mitapivat, an oral allosteric activator of pyruvate kinase, has emerged as a promising targeted therapy [11]. Clinical trials have demonstrated improvement in hemoglobin levels and reduction in transfusion burden among adults with PKLR mutations responsive to enzyme activation [11]. However, pediatric experience remains limited, and further studies are required before routine use in infancy can be recommended [5,11]. Other disease-modifying therapies like gene therapy continue to advance the clinical management framework for PKD [12].

This case expands the mutational spectrum of PKLR by describing a novel c.1376C>G (p.Ala459Gly) variant associated with a phenotype consistent with severe PKD. Although functional validation is lacking, the clinical findings highlight the importance of reporting novel variants to improve genotype-phenotype correlations and facilitate future assessment of their clinical significance.

## Conclusions

We report a severe infantile presentation of transfusion-dependent hemolytic anemia in a patient harboring a novel homozygous PKLR variant, c.1376C>G (p.Ala459Gly), with early iron overload and reduced G6PD enzyme activity detected by enzymatic assay. Although the clinical phenotype is compatible with PKD, the identified PKLR variant is currently classified as a variant of uncertain significance, and its pathogenicity cannot be confirmed based on this single case. The reduced G6PD enzyme activity may have contributed to the clinical severity. This case highlights the importance of comprehensive clinical, biochemical, and molecular evaluation in hereditary hemolytic anemias, particularly in consanguineous populations. Further functional studies and additional reports are needed to clarify the clinical significance of this novel PKLR variant.
